# A high-content screen reveals new regulators of nuclear membrane stability

**DOI:** 10.1038/s41598-024-56613-1

**Published:** 2024-03-12

**Authors:** Amanda L. Gunn, Artem I. Yashchenko, Julien Dubrulle, Jodiene Johnson, Emily M. Hatch

**Affiliations:** 1 Divisions of Basic Sciences and Human Biology, The Fred Hutchinson Cancer Center, Seattle, WA USA; 2 Cellular Imaging Shared Resource, The Fred Hutchinson Cancer Center, Seattle, WA USA; 3 Molecular and Cellular Biology Graduate Program, University of Washington, Seattle, WA USA

**Keywords:** Nuclear envelope, Nuclear pore complex

## Abstract

Nuclear membrane rupture is a physiological response to multiple in vivo processes, such as cell migration, that can cause extensive genome instability and upregulate invasive and inflammatory pathways. However, the underlying molecular mechanisms of rupture are unclear and few regulators have been identified. In this study, we developed a reporter that is size excluded from re-compartmentalization following nuclear rupture events. This allows for robust detection of factors influencing nuclear integrity in fixed cells. We combined this with an automated image analysis pipeline in a high-content siRNA screen to identify new proteins that both increase and decrease nuclear rupture frequency in cancer cells. Pathway analysis identified an enrichment of nuclear membrane and ER factors in our hits and we demonstrate that one of these, the protein phosphatase CTDNEP1, is required for nuclear stability. Analysis of known rupture determinants, including an automated quantitative analysis of nuclear lamina gaps, are consistent with CTDNEP1 acting independently of actin and nuclear lamina organization. Our findings provide new insights into the molecular mechanism of nuclear rupture and define a highly adaptable program for rupture analysis that removes a substantial barrier to new discoveries in the field.

## Introduction

Developing mechanistic insight into nuclear membrane rupture is crucial to understanding this potential driver of cancer progression and metastasis, however technological barriers limit our ability to perform broad unbiased screens and define molecular mechanisms. Nuclear membrane rupture is defined as the rapid loss of nucleus compartmentalization in interphase due tension-induced hole formation in the outer and inner nuclear membranes. These ruptures result in the mixing of nuclear and cytoplasmic proteins and organelles^1–3^. Ruptures are typically repaired within minutes with limited effects on cell proliferation or viability^4,5^. However, they can cause or enhance DNA damage, gene expression changes, and activation of cell invasion and inflammation pathways, especially under conditions of mechanical stress^2,4–9^. In addition, persistent rupture of micronuclear membranes can lead to catastrophic changes in chromosome structure, ongoing chromatin defects, and altered gene expression patterns that are proposed to be initiating events in some tumors and drivers of metastasis^10–15^.

Nuclear membrane rupture occurs in a range of mechanically challenging conditions in vivo*,* including migration through dense extracellular matrices, at the leading edge of tumors, and during nuclear migration in fission yeast, *C. elegans*, and mouse models of laminopathies^4,5,8,16–22^. Changes in nuclear membrane stability underlie pathogen infection, neurodegeneration, autoimmune diseases, cancer progression, and laminopathy pathologies^23,24^, suggesting nuclear envelope dynamics must be strictly regulated for proper cell functioning.

A current model of nuclear membrane rupture is that it occurs in response to an imbalance between force and force resistance at the nuclear envelope^25–27^. In general, membrane rupture occurs when a gap forms in the nuclear lamina matrix, exposing the membrane to cellular forces. This loss of support is frequently followed by membrane blebbing and the simultaneous disruption of the inner and outer nuclear membranes^24,28–31^. Actomyosin forces are a major driver of nuclear rupture in tissue culture and migrating cells. In cultured cells, both increased nuclear compression by perinuclear-actin cables and increased actomyosin contractility have been shown to drive rupture^32–37^. Opposing these forces, heterochromatin and nuclear lamina proteins provide mechanical resistance and limit nuclear lamina gap frequency, membrane blebs, and rupture^3,30,38–42^. Despite the elucidation of this general model, many questions about rupture remain, including how lamina gaps form, what triggers the transition from membrane expansion to membrane rupture, and how the frequency and size of the rupture affects the consequences of losing nucleus compartmentalization.

The current gold standard for identifying and quantifying nuclear membrane rupture is live-cell imaging of a fluorescent protein tagged with a nuclear localization signal (NLS). Although software exists to automatically quantify ruptures from these data^40^, the sheer size of the data and imaging time required precludes systematic identification of membrane stability regulators using live-cell imaging methods. Several proteins remain at rupture sites for some time after membrane repair, including cGAS, BAF, and lamin A, and have been used to identify ruptures in fixed tissue^21^. However, the extent and duration of their localization can be highly variable and dependent on additional factors^14,43^, limiting their utility as a screening tool. An alternative approach uses mislocalization of large complexes to the nucleus or the cytoplasm to identify cells that have previously lost nuclear integrity. During rupture soluble proteins and small organelles diffuse through the membrane gap and become mislocalized^2,3,26,44^. Chromatin-directed nuclear re-compartmentalization initiates within seconds of the chromatin being exposed to the cytoplasm and proteins containing nucleus and cytoplasmic sorting signals (NLS and NES, respectively) are rapidly relocated^4,33,45,46^. In contrast, organelles and large proteins lacking sorting signals, including Hsp90, 53BP1, and PML bodies, can persist in the wrong compartment for several hours^8,25,44,47,48^.

Building on these observations, we developed a reliable and well-tolerated reporter of nuclear membrane rupture that can be used in fixed cells, called RFP-Cyto. We demonstrate that mis-localization of RFP-Cyto to the nucleus is a rapid, precise, and durable marker of nuclear membrane rupture. We describe an automated analysis pipeline to quantify rupture frequency in fixed cell populations using this reporter and combine it with siRNA screening to discover 22 new regulators of rupture in a high-throughput and unbiased manner. Co-analysis of GFP-NLS localization allowed us to further expand the rupture phenotypes under investigation to include proteins that regulate membrane repair or single cell rupture frequency and identified the kinase STK11 as an additional stability factor. We apply a suite of imaging and analysis tools to validate the nuclear lipin phosphatase CTDNEP1 as a significant rupture inhibitor and delineate its effects on cellular structures associated with nuclear stability. We demonstrate that CTDNEP1 loss does not substantially alter nuclear lamina gap frequency, NPC density, or nuclear confinement by perinuclear actin, suggesting that it acts in an alternative mechanism of membrane stabilization. This work represents the first systematic identification of nuclear membrane rupture and repair factors in human cells and defines a set of tools to rapidly identify factors acting in new pathways. Our tools substantially reduce the barrier to identifying cellular changes that impact nuclear membrane stability and pave the way for future genome-wide analyses.

## Results

### RFP-Cyto is size excluded from re-compartmentalization following nuclear rupture

**Figure 1:**
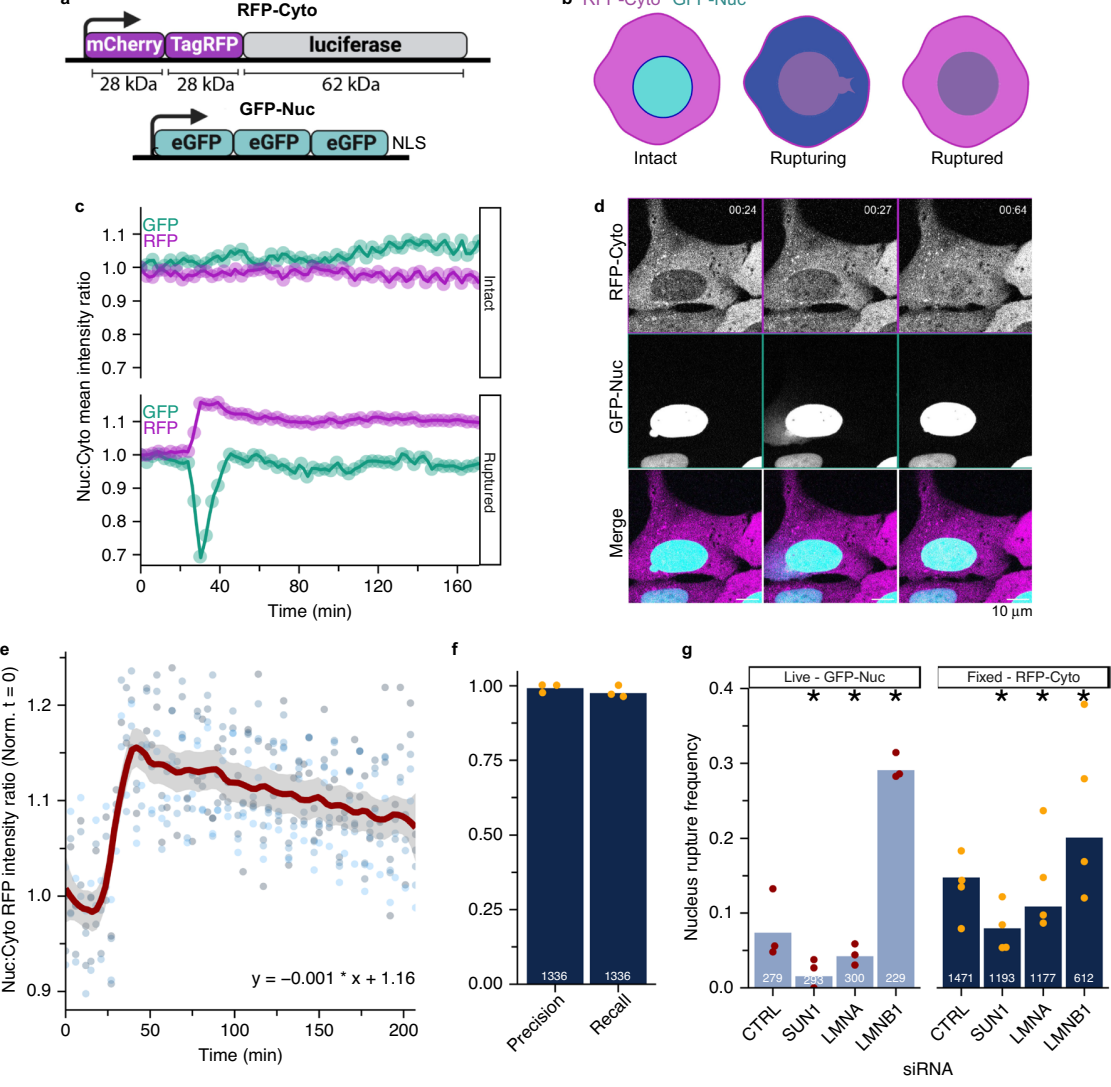
RFP-Cyto is size excluded from re-compartmentalization following nuclear rupture, enabling fixed cell analysis. (**a**) Schematic of RFP-Cyto and GFP-Nuc reporters. (**b**) Schematic of rupturing cells with incorporated reporters. (**c**) Representative traces of RFP-Cyto and GFP-Nuc Nuc:Cyto intensity ratios in a cell with an intact nuclei or a rupturing nuclei. (**d**) Representative image panel of cells expressing rupture reporters in (a) before, during, and after nucleus rupture. (**e**) Linear model of Nuc:Cyto RFP-Cyto ratio before, during, and after a single rupture with best fit line to descending slope. n = 5. Loess smoothing = 0.2, standard error = gray line, dots = individual datapoints. (**f**) Precision and recall comparing ruptures manually quantified using RFP-Cyto versus GFP-Nuc mislocalization by live-cell imaging in the same cell populations, U2OS RuptR cells treated with siRNAs indicated in (**g**). N = 3. (**g**) Manual nuclear rupture frequency analysis for live cells based on GFP-Nuc imaging (N = 3) and fixed cells based on RFP-Cyto nuclear localization (N = 4). *p < 0.05, Barnard’s test. Cells: U2OS RuptR. For all graphs: bars = pooled proportions, dots = individual replicates, pooled n values indicated on graph.

To develop a fixed cell reporter for interphase nuclear rupture, we took advantage of the fact that large objects and proteins lacking an NLS or NES remain mislocalized after nuclear membrane rupture and repair. We found that a construct containing luciferase tagged with two RFPs was the smallest one (118 kD) which showed exclusive cytoplasm localization during interphase (RFP-Cyto, Fig. 1A, Fig. S1A). We optimized for a minimally sized reporter to increase the likelihood that small, short ruptures would result in reporter mislocalization to the nucleus. In contrast to proteins tagged with an NLS (e.g. GFP-Nuc), we expected RFP-Cyto would remain mislocalized after membrane repair (Fig. 1A,B).

We stably expressed RFP-Cyto in U2OS cells expressing GFP-Nuc and an shRNA against LMNB1 and monitored RFP-Cyto localization during nuclear rupture by live-cell imaging. Constitutive expression of LMNB1 shRNAs in U2OS cells leads to a modest reduction in protein levels but substantially increases nuclear rupture frequency and allows for sensitive identification of in vivo relevant mechanisms^35,46,49^. We imaged this cell line, U2OS RuptR, every 3 min for 24 h and analyzed both GFP and RFP mean nuclear intensities during rupture events. As expected, RFP-Cyto intensity increased in the nucleus after rupture, identified by the rapid decrease in GFP-Nuc signal, and remained mis-localized after membrane repair, indicated by GFP-Nuc fluorescence recovery. In the absence of nuclear rupture, no sustained change in RFP-Cyto nuclear intensity was observed (Figs. 1C,D, S1B, Movie S1), with some variation in the size of the ratio change likely attributable to differences in initial fluorescence intensities and rupture extent and frequency. RFP-Cyto persisted in the nucleus for at least 150 min after rupture, and nuclei with one or more ruptures often maintained elevated nuclear RFP to the end of imaging (Figs. 1E, S1B). Importantly, RFP-Cyto was not mislocalized to post-mitotic nuclei, showing recovery of the Nuc:Cyto ratio soon after nuclear envelope assembly (peak GFP-Nuc Nuc:Cyto intensity)^50,51^ (Fig. S1C, Movie S2).

To determine the accuracy and sensitivity of RFP-Cyto as an interphase rupture reporter, we quantified RFP-Cyto mislocalization compared to GFP-Nuc mislocalization after transfection with siRNAs that have been shown to increase rupture frequency (LMNB1), decrease rupture frequency (Sun1), or have no effect (LMNA) in this cell line^38^. Protein loss was validated by Western blot (Fig. S1D). First, we compared RFP-Cyto and GFP-Nuc mislocalization during live-cell imaging in all conditions and found that RFP-Cyto nuclear localization accurately defined nuclear rupture events, with a pooled precision of 99% and recall of 99% (Fig. 1F). Rare ruptures missed by RFP-Cyto tended to be short and in cells with low RFP-Cyto expression (Movies S3–S5). Analysis of individual siRNA nuclear rupture frequency by live-cell imaging of GFP-Nuc found that, as expected^35,38^, lamin B1 loss increased rupture and Sun1 loss suppressed it (Fig. 1G, left). Although lamin A/C depletion can increase rupture frequency^2,40^, we did not see a consistent increase upon lamin A/C loss, in line with previous results from us and others^31,37^. Importantly, similar results were observed when ruptures were analyzed by assessing RFP-Cyto localization to the nucleus in fixed cells (Fig. 1G, right). Overall, we found that RFP-Cyto tends to find higher rupture frequencies compared to live-cell imaging of GFP-Nuc, likely because it samples a longer time period (time from last mitosis versus 24 h), but that it has increased variability, likely due to its higher sensitivity to reduced fluorescence intensity, fixation conditions, and focal plane changes. These data demonstrate that analysis of fixed U2OS RuptR cells provides accurate information about relative rupture frequencies with a high degree of sensitivity.

### An automated analysis pipeline accurately detects alterations to nuclear stability in U2OS RuptR cells

**Figure 2:**
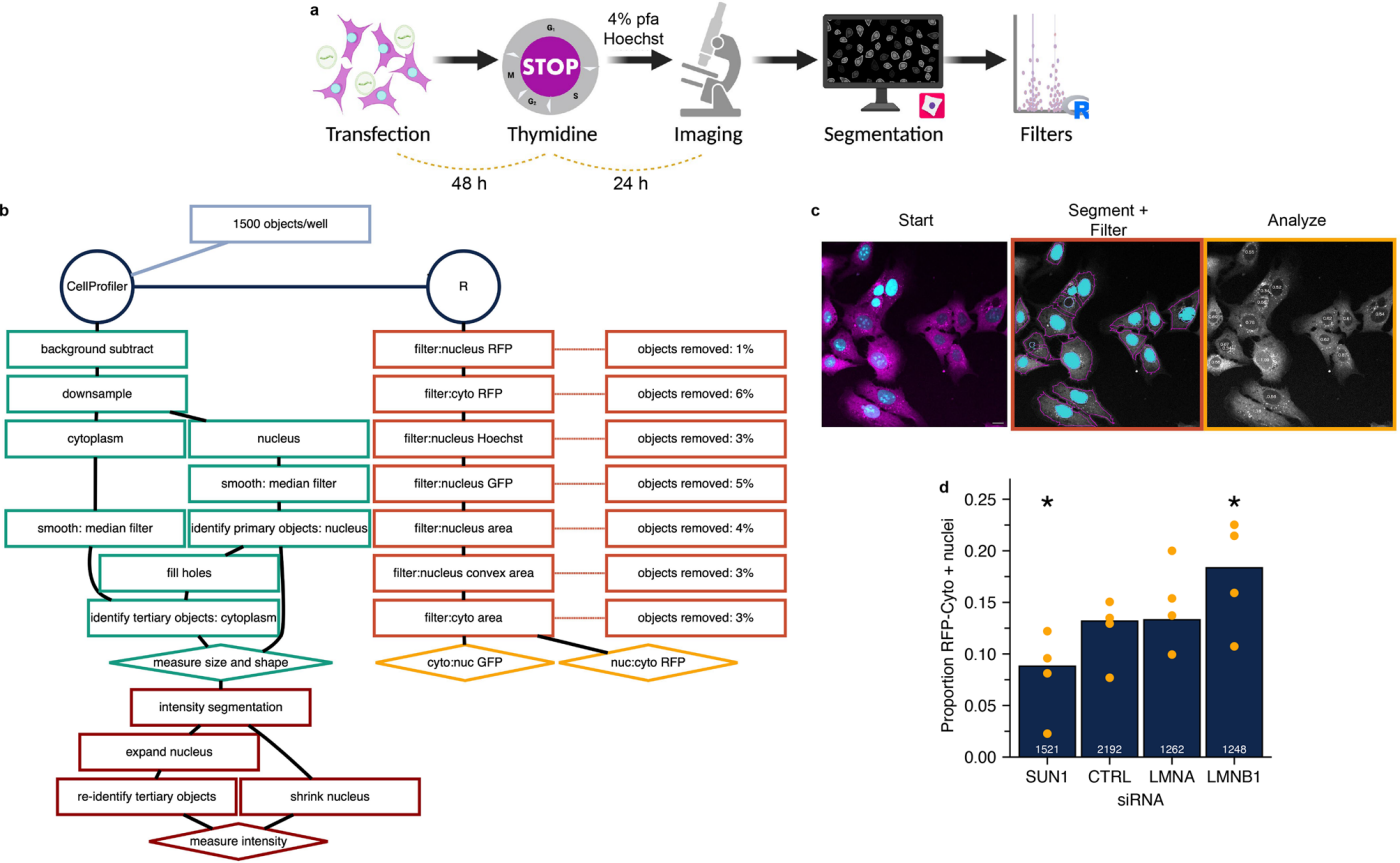
An automated bioinformatics pipeline allows accurate fixed cell analysis of rupturing nuclei. (**a**) Schematic of experimental pipeline (**b**) Detailed schematic of data processing steps. (**c**) Representative images of one field of view. Left: start image, center: false colored nuclei passing filtration, right: overlay with nuc:cyto RFP values. (**d**) Proportion of cells with RFP-Cyto Nuc:Cyto ratios above the threshold from cells transfected with positive or negative control siRNA pools and analyzed using pipeline. Compare to Fig. 1g, fixed. Cells: U2OS RuptR, Stats: Table S2, *p < 0.05, Barnards test. N = 4.

To enable high throughput analysis of RFP-Cyto localization and intensity, we developed a segmentation strategy in CellProfiler^52^ coupled with a set of post-segmentation intensity and morphological filters in R to remove contaminating objects. This workflow was developed on U2OS RuptR cells transfected with siRNA, arrested in S phase for 24 h to enhance rupture frequency and reduce mitotic false positives^35^, fixed, and labeled with Hoechst to mimic screening conditions (Fig. 2A). Nuclei and cells were segmented in CellProfiler based on Hoechst and RFP-Cyto signal, respectively, and morphological parameters were analyzed (Fig. S2A, Fig. 2B,C). Objects were then shrunk to limit overlapping signal or segmentation errors and RFP, GFP and Hoechst mean intensity in the cytoplasm and nucleus were measured (Fig. 2B, Fig. S2A). Intensity and morphology filters were applied post-processing to eliminate data from cells with low RFP-Cyto expression, missegmented objects, mitotic or dead cells, and out of focus cells. Mitotic cells were removed by setting a maximum Hoechst intensity threshold, coupled with a maximum solidity threshold, and out of focus cells were reduced by setting a minimum Hoechst intensity threshold. Cells undergoing cell death were limited by setting minimum nuclear size and solidity thresholds (Fig. S2B). Filter thresholds were set globally for intensity measurements and per condition for morphology measurements to accommodate expected variations in nucleus shape and size during screening (Fig. 2B, Fig. S2A). After filtering, over 75% of cells were retained and RFP-Cyto localization was determined for these by calculating the nucleus to cytoplasm (Nuc:cyto) mean intensity ratio.

To validate this pipeline, U2OS RuptR cells were reverse transfected with pooled siRNAs targeting LMNB1, SUN1, and LMNA. Following knockdown for 48 h, cells were arrested in S phase for a further 24 h before fixation. The Nuc:cyto RFP-Cyto mean intensity ratio was calculated on cells passing all filters for each condition. The threshold for ruptured nuclei (RFP-Cyto +) was defined as a Nuc:cyto RFP ratio greater than one standard deviation above the median of the CTRL siRNA well for each technical replicate. Comparing the proportion of cells with ruptured nuclei between siRNA conditions replicated the significant changes in rupture frequency previously identified by manual analysis of fixed and live-cell populations (Fig. 2D, compare to Fig. 1G). These results demonstrated that our screen and image analysis pipeline design was sufficiently sensitive to identify biologically relevant changes in rupture frequency.

### A high content screen identifies new factors influencing nuclear integrity

To identify new regulators of nuclear membrane rupture, we applied this pipeline to a plate-based siRNA screen targeting known categories of nuclear membrane stability regulators (49%) and randomly selected genes (51%) (Table S9). Targeted categories, each of which were enriched overall within the screen (Fig. S4A), included chromatin organization^30^, tumor suppressors^53–55^, nuclear envelope localized^2,3,31,40,56^, ER/nuclear membrane structure^57^, and cytoskeleton^35,37,58,59^. Images from at least three technical replicates were analyzed and the proportion of cells with ruptured nuclei (RFP-Cyto +) was calculated for each replicate. The RFP-Cyto Nuc:Cyto ratio threshold for rupture was calculated for each replicate based on the pooled median of the three control siRNA wells. To determine the robustness of our image analysis pipeline, images from siRNA conditions with statistically significant changes in nucleus size and solidity (Fig. S3A) or in median RFP-Cyto intensity were manually evaluated for segmentation and RFP-Cyto + errors. Our automated analysis and filtering was robust to each of these parameters, with a minimum precision of 80% and a recall of 94% and a consistently high frequency of proper cell and nucleus segmentation (Fig. S3B,C). Visual inspection also led to the exclusion of PSMC1 knockdown, an outlier in fluorescence intensity, from further analysis due to excessive cell death, consistent with its function as an essential proteosome component^60^ (Fig. S3D).

**Figure 3:**
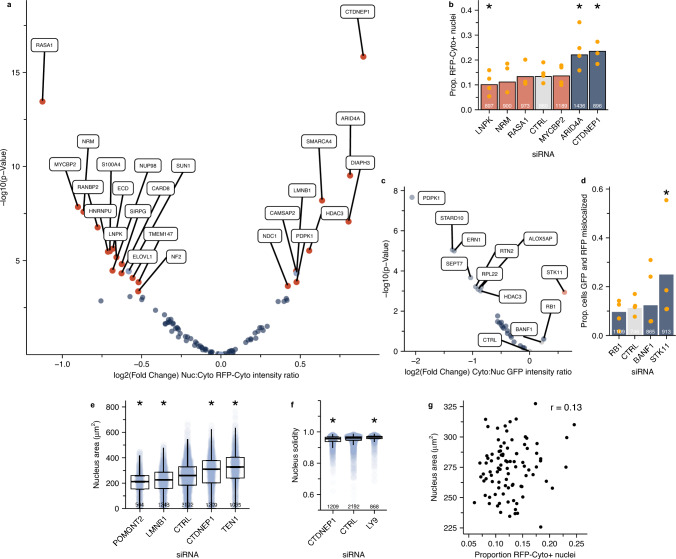
A high content siRNA screen accurately identifies factors influencing nuclear integrity and morphology. (**a**) Volcano plot of siRNA pools that significantly altered nuclear rupture frequency determined by proportion cells with RFP-Cyto Nuc:Cyto ratio over threshold compared to control wells. Labels = p < 0.05 by Bonferroni adjusted Barnards test. (**b**) Analysis of nucleus rupture frequency in fixed cells after single siRNA transfection against indicated target. *p < 0.05, Barnards test. Bar color indicates effect direction in screen. N = 4. (**c**) Volcano plot of siRNA pools that significantly altered rupture kinetics determined by proportion of RFP-Cyto positive nuclei where GFP-Nuc Cyto:Nuc ratio was also over threshold compared to control wells. Only conditions where >  = 175 RFP + nuclei were present in each replicate were analyzed. BANF1 is a positive control. Labels indicate control siRNAs and targets that increased GFP-Nuc mislocalization (p < 0.05, Barnards test). N = 4. (**d**) Validation of GFP-Nuc screen hits in fixed cells transfected with single siRNAs against indicated target. 1/1 hit from screen significantly increased GFP-Nuc mislocalization to cytoplasm. * = p < 0.05, Barnards test. N = 3–4. (**e**) Analysis of nuclear area from conditions in (**a**) with largest median difference from control median. (**f**) Analysis of nucleus solidity from conditions in (**a**) with largest median difference from control median. **g.** Median nucleus area did not correlate with increased rupture frequency across conditions tested in (**a**) (Pearson). (**e**–**g**): *p < 0.05, Wilcoxon test, N = 4. Cells: U2OS RuptR, Stats: Tables S3–S8.

Our screen successfully identified 22 genes as hits (24% of screen), with 14 (15%) decreasing rupture frequency when depleted and 8 (8.6%) increasing it, based on an adjusted p-value cut-off of 0.05 (Barnard’s test, Bonferroni correction for multiple testing) and a log_2_ fold-change cut-off of 0.3 (Fig. 3A, Fig. S3E, Table S3). To validate our screen results, we selected six genes to re-analyze using single siRNAs. siRNA efficacy was validated by qRT-PCR (Fig. S3F). Nuc:cyto RFP-Cyto intensity ratios were analyzed using the same experimental design as the screen. We found 3/6 targets significantly altered the proportion of ruptured nuclei in the same direction as the screen (Fig. 3B), demonstrating that our screen allows accurate and unbiased identification of nuclear membrane stability factors that both stabilize and destabilize the membrane.

We further extended our screen to quantify conditions that alter the proportion of rupturing, versus ruptured, nuclei (Fig. 1B) to identify proteins that inhibited membrane repair and/or increased single cell rupture frequency. The proportion of RFP-Cyto + cells where GFP-Nuc was also mislocalized to the cytoplasm, defined as a Cyto:Nuc GFP mean intensity greater than one standard deviation from the median of the three siCTRL populations, was calculated for each condition with ≥ 175 RFP-Cyto + nuclei per replicate. As a positive control, we depleted BANF1 (BAF), which is required for efficient membrane repair^45,46^ (Fig. S3G). Of the 26 screened siRNAs with sufficient rupture events, only depletion of RB1 and STK11 increased the frequency of RFP-Cyto GFP-Nuc double positive cells at or above the level of BANF1 (Fig. 3C). To validate these hits, BANF1, RB1, and STK11 were depleted in U2OS RuptR cells by single siRNA transfection (Fig. S3F,G) and the proportion rupturing cells was compared to cells transfected with control siRNAs. Both BANF1 and STK11 depletion increased the proportion of rupturing nuclei, with STK11 significantly higher than control (Fig. 3D). These results strongly suggesting that STK11, a cancer-associated kinase that acts as a master regulator of cell growth and metabolism^61^, has additional functions in maintaining nuclear integrity. Unexpectedly, several conditions significantly reduced the frequency of rupturing nuclei (Fig. 3C). Loss of these genes could potentially identify conditions that limit nuclear rupture extent or accelerate repair. However the large size of this group suggests that more analysis is required to determine their accuracy.

Changes in nucleus morphology can be strongly correlated with changes in nucleus stability^62^. Our analysis of nuclear area and nucleus solidity identified five conditions that significantly altered nuclear size or lobulation, including LMNB1, a known nuclear growth factor^63,64^, and CTDNEP1, a known nuclear size restriction factor^65^ (Fig. 3E,F). Despite having opposite effects on nuclear size, both CTDNEP1 and LMNB1 depletion increased nuclear rupture, and Pearson correlation analysis found no significant relationship between nucleus rupture and median nucleus area in the screened genes (Fig. 3G).

**Figure 4:**
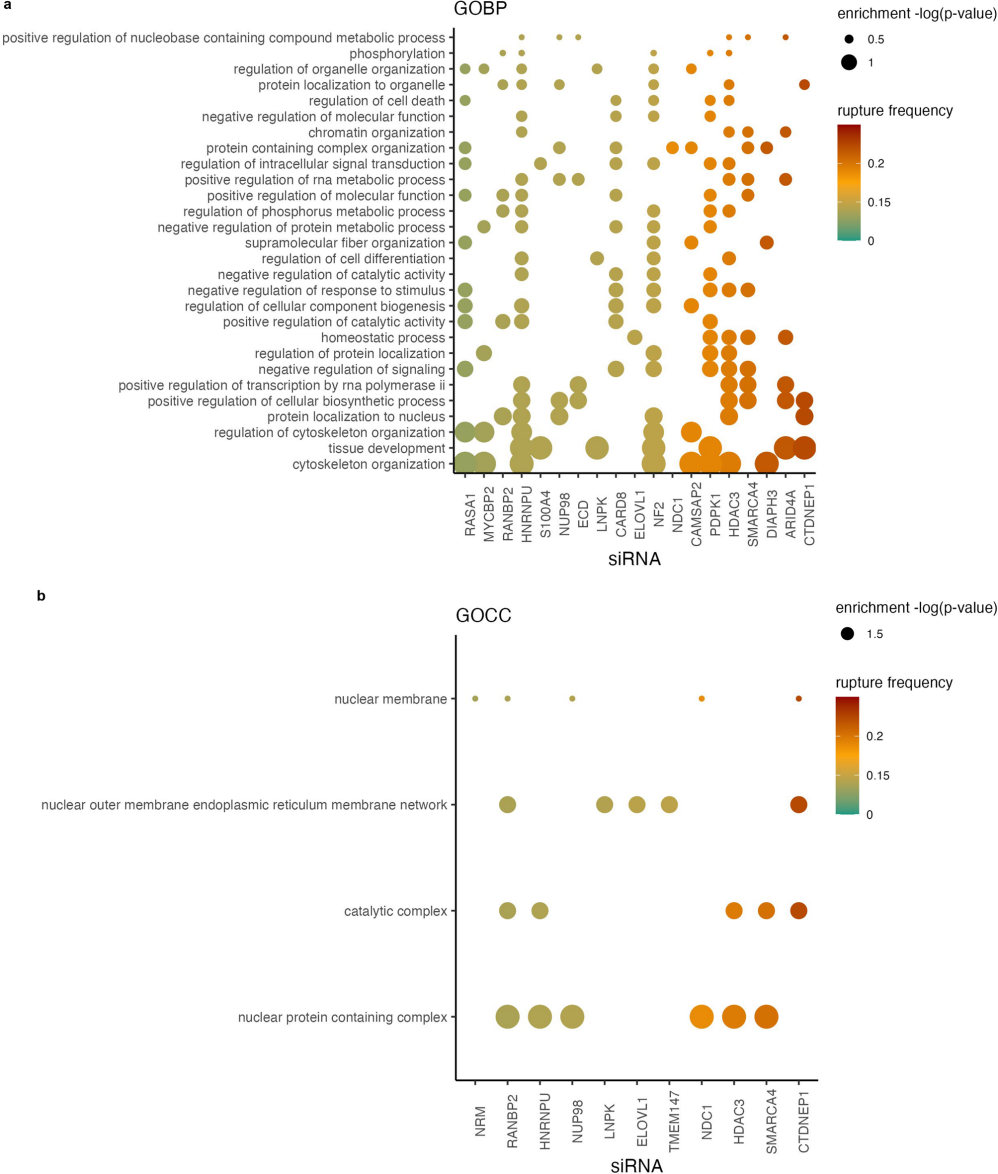
Hypergeometric enrichment identifies nuclear and cellular pathways influencing nuclear integrity. (**a**,**b**) Hypergeometric enrichment of gene ontology terms (**a**: GOBP, **b**: GOCC) for siRNAs with significant RFP-Cyto screen results. Rupture frequency indicated by color, p-value indicated by size. Stats: Tables S10 and S11.

To determine whether screen hits were enriched in specific cellular pathways, we performed GO (gene ontology) analysis with hypergeometric enrichment statistics to correct for the bias towards nuclear factors in our gene set (Fig. S4A). Overall, our hit list was only slightly enriched in targeted compared to randomly selected genes (Fig. S4B). GO term analysis identified a high enrichment in predicted processes, including cytoskeletal organization and nuclear protein complexes, as well as unexpected processes and components, including tissue development and the outer nuclear membrane /ER network (Fig. 4). Although membrane shaping proteins were a targeted gene class, their enrichment over proteins in the inner nuclear membrane and NPC strongly suggests that these factors are a new key regulator of nuclear stability.

### CTDNEP1 maintains nuclear membrane stability in interphase

**Figure 5:**
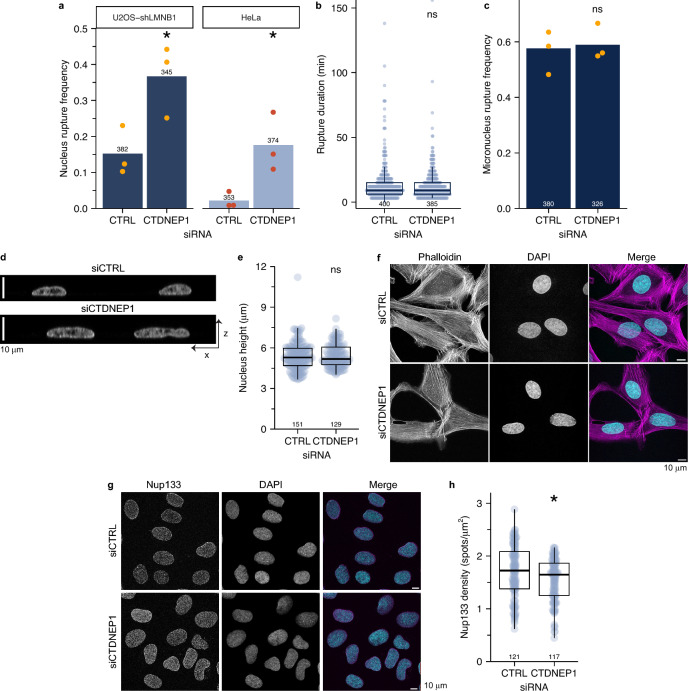
CTDNEP1 loss increases rupture frequency in the absence of NPC or confinement defects. (**a**) CTDNEP1 depletion by single siRNAs increases nucleus rupture frequency in U2OS-shLMNB1 RFP-NLS and HeLa GFP-Nuc cells compared to control, analyzed by live-cell imaging. (**b**) CTDNEP1 depletion does not significantly increase rupture duration, analyzed by live-cell imaging in U2OS-shLMNB1 RFP-NLS. (**c**) Micronucleus rupture frequency analyzed in fixed asynchronous cells induced to form micronuclei via spindle assembly checkpoint inhibition. (**d**) Representative DAPI orthosections of cells treated with indicated siRNAs showing similar nuclear height profiles. (**e**) Quantification of nuclear height after single siRNA transfections shows no significant change after CTDNEP1 depletion. (**f**) Representative images of actin organization (phalloidin) in siRNA treated cells. Max. intensity projections of cell top half. (**g**) Representative images of NUP133 foci. Max. intensity projection of nucleus top half in siRNA-treated cells. (**h**) NUP133 quantification showing decreased foci density following CTDNEP1 depletion. Cells: U2OS GFP-Nuc, unless indicated. Stats: Tables S14–S18. For all graphs, N = 3, *p < 0.05, ns = p > 0.05. For (**a**) and (**c**): Barnards test. For (**b**,**e**,**h**): K-S test.

The enrichment of ER/nuclear membrane factors in our screen hits prompted us to further characterize the function of one of these proteins, CTDNEP1, in nuclear membrane stability. CTDNEP1 is a protein phosphatase that localizes to the nuclear membrane and regulates lipid composition through activation of the phosphatidic acid phosphatase, lipin1^66^. CTDNEP1 caused the largest increase in nuclear rupture upon depletion in our screen (Fig. 3A) and additional validation with a second siRNA and qRT-PCR confirmed the depletion of CTDNEP1 and the specificity of the nuclear rupture phenotype for CTDNEP1 loss (Fig. S5A,B). As a positive control for CTDNEP1 depletion, we examined ER area and nuclear size and found that both were increased, as expected, in our cells^65,67^ (Fig. 3E, Fig. S5C–E). Live-cell imaging analysis of CTDNEP1 siRNA depletion in U2OS shRNA-LmnB1 and HeLa cells expressing GFP-Nuc caused a significant increase in rupture frequency in both cell lines, further validating our fixed-cell analysis and demonstrating that this effect is not cell type specific nor dependent on lamin B1 depletion (Fig. 5A, Movie S6). CTDNEP1 loss can delay nuclear membrane resealing after division^65^, therefore we analyzed rupture duration by live-cell imaging of GFP-Nuc in U2OS RuptR cells. We found no statistical difference between control and CTDNEP1 depletion (Fig. 5B), consistent with the results of our fixed-cell GFP-Nuc screen, suggesting that a requirement for CTDNEP1 in membrane closure is context dependent. CTDNEP1 loss did not significantly increase micronucleus rupture (Fig. 5C), suggesting that CTDNEP1 is an inhibitor of membrane rupture specific to the main nucleus.

We next sought to determine the mechanism by which CTDNEP1 stabilizes the nuclear membrane. Analysis of nuclear height found no significant difference between CTDNEP1 and control depleted cells (Fig. 5D,E) and no substantial changes were observed in perinuclear actin organization (Fig. 5F), strongly suggesting that CTDNEP1 loss does not increase nucleus confinement. We next quantified NPC density as changes in nuclear transport have been linked to increased nuclear membrane tension^36^ and CTDNEP1 has functions in NPC formation^68^. Depletion of CTDNEP1 caused a slight, but significant, decrease in NPC density, as determined by Nup133 labeling (Fig. 5G,H), suggesting that CTDNEP1 loss is unlikely to increase nuclear rupture via nuclear transport changes.

**Figure 6:**
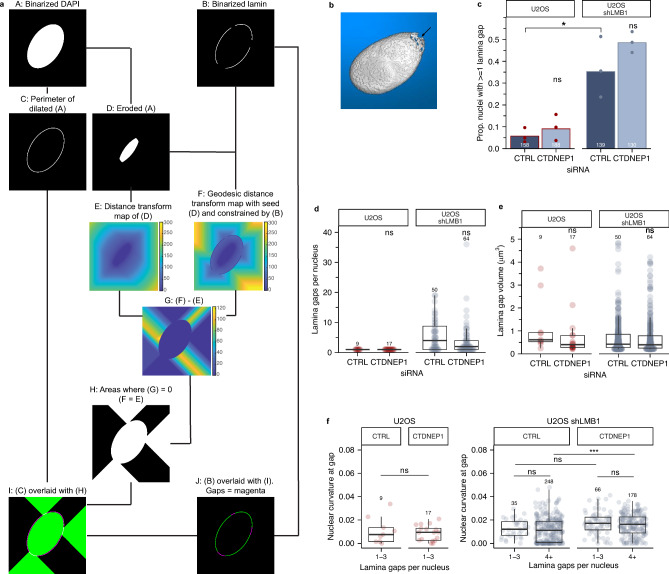
Automated quantification of nuclear lamina gaps identifies minimal increase in lamina gap frequency from CTDNEP1 loss. (**a**) Overview of the image processing steps used to identify and characterize gaps in the nuclear lamina, full description available in methods. Briefly, areas of equivalence between a cartesian distance map of the nucleus and a lamina-constrained map of the same are projected onto the nucleus perimeter. Putative gaps are then filtered for size and intensity to identify true gaps. (**b**) Rendering of the nuclear lamina (lamin A) after lamina gap analysis with one gap indicated by arrow. (**c**–**e**) Characterization of lamina gaps in U2OS GFP-Nuc or U2OS shLMNB1 2 × RFP-NLS cells transfected with indicated siRNAs and labeled with lamin A. Proportion of nuclei with at least one gap (**c**), gaps per nucleus with at least one gap (**d**), and gap size (**e**) are quantified. * = p < 0.05, ns = p > 0.05, Barnard’s test (**c**), or Kruskal–Wallis (K–W) test (**d**,**e**). (**f**) Curvature of nucleus at nuclear lamina gaps quantified for siRNA treated cells. Nuclei binned into low gap (1–3) or high gap (4 +) categories. Lamina gaps are present on flat nuclear surfaces when gap number is high. This localization is reduced by CTDNEP1 depletion. ns = p > 0.05, K-S or K-W test. ***p < 0.001, K-W test. Stats: Tables S20–S22. N = 3 for all quantifications.

A major predictor of nuclear membrane rupture in both nuclei and micronuclei is the presence of nuclear lamina gaps. To robustly and sensitively quantify nuclear lamina gaps, we developed an application that uses distance transformation on binarized images of DAPI and a nuclear lamina protein, here lamin A, to automatically define the presence, number, and size of the nuclear lamina gaps per nucleus, as well as the relative curvature of the nucleus at gap locations (Fig. 6A,B). To validate this method, we compared nuclear lamina gaps in S-phase arrested U2OS cells with or without shRNAs against LMNB1, which significantly increases gap number^35^. As expected, LMNB1 depletion significantly increased the number of nuclei with nuclear lamina gaps and the gap number per nucleus (Fig. 6C,D, CTRL siRNA), but not lamina gap size (Fig. 6E). Lamina gaps are frequently located at the highly curved poles of the nucleus^25,69^. Our analysis confirmed that gaps are enriched on curved surfaces in both cell lines when few gaps are present (1–3), but also showed an increased localization to flat nuclear surfaces (curvature = 0 μm^−1^) as the gap number increases (Fig. 6F). Together these data strongly suggest that depletion of lamin B1 alters the frequency, but not the physical characteristics, of nuclear lamina gaps and validated our automated approach. We next applied this analysis to cells depleted of CTDNEP1 and found a small increase in the proportion of nuclei with nuclear lamina gaps and no increase in gap number or size in both U2OS and U2OS shLMNB1 cells (Fig. 6C,E). The limited and inconsistent increase in cells with nuclear lamina gaps, and the lack of change in gap frequency, unlike after lamin B1 depletion, suggest that increased lamina disruption is unlikely to fully explain the ~ 2.5 fold increase in rupture frequency upon CTDNEP1 depletion (Fig. 6A). Unexpectedly, CTDNEP1 loss reduced the frequency of gaps on flat nuclear surfaces (Fig. 6F). A partial explanation may be the increased mean curvature of CTDNEP1 depleted nuclei (Figs. 3F, S3A), but orthosection and height analyses indicate that CTDNEP1 loss does not eliminate flat nuclear surfaces (Fig. 5D,E), suggesting that another mechanism is driving lamina gaps clustering at the poles in this condition. Together, our results strongly suggest that CTDNEP1 acts in part through exacerbating existing nuclear lamina defects but also through an independent mechanism of nuclear membrane rupture.

## Discussion

We have developed a new screening pipeline, based on automated image analysis of a nuclear-excluded reporter, that enables rapid identification of conditions that impact nuclear stability without the need for live-cell imaging. This system allows us for the first time to define novel cellular regulators of nuclear membrane rupture in an unbiased manner. In addition to paving the way for future large-scale screens, our fixed-cell technique will accelerate analysis of molecular mechanism experiments in this field and enable characterization of nuclear rupture in samples that are not amenable to long-term live-cell imaging, including rapidly moving cancer cells and 3D organoids. We expect this technology will be adaptable to any cell type that permits exogenous protein expression and has a cytoplasm compartment that can be segmented. In addition, this study defines a suite of automated image analysis methods, including a new open-source application for nuclear lamina gap quantification, that can be employed in a secondary screen to place hits in functional pathways based on their effects on nuclear structure. By creating standardized methods for these analyses, our tools will also facilitate comparison of nuclear structures across systems and enable detailed molecular analysis of mechanisms that disrupt the nuclear lamina. Overall, our new reporter system lowers the barrier of entry for biologists in any field to evaluate the contribution of nuclear membrane rupture to their phenotype of interest and quantitatively identify changes in contributing cellular structures to accelerate mechanistic dissection.

Nuclear lamina gaps have been observed in both developmental and disease contexts in a broad group of species^24,70^. They have been the subject of much modeling and analysis^33,69,71–73^, yet fundamental questions about them remain, including how they form, how long they persist, and whether they can be repaired. One limiting factor in generating these models is the lack of large quantitative datasets. The nuclear lamina analysis application developed in this study overcomes this challenge by providing robust and rapid quantification of many gap parameters, including number, size, solidity, and nuclear curvature from 3D fluorescence images. Additional nuclear morphometric parameters are included in the output including height, volume, surface area, and label intensity. Our initial analysis of the number, size, location of lamina gaps in U2OS cells with and without lamin B1 depletion strongly suggests that gaps form and expand by the same mechanism in both conditions, but that the initial barrier to forming a gap is lowered by decreased lamin B1. Similar analyses of other conditions increasing nuclear lamina gap frequency may further stratify these phenotypes and reveal new mechanisms.

Our initial siRNA screen against targeted and randomly selected genes had a high hit and validation rate for both groups, strongly suggesting that nuclear rupture occurs in many more conditions than are currently known. In addition, our results suggest that changes in nuclear membrane stability is a sensitive assay to detect alterations in nuclear or cytoskeletal structure and that our system could be a valuable tool for uncovering new regulators of both. This screen also identified proteins that promote nuclear membrane rupture in cancer cells. Future analysis of how these proteins act will provide new insight into how cells can adapt to high rupture rates and their depletion will enable critical experiments defining the specific consequences of membrane rupture in cells with disrupted nuclear structures. One caveat of this screening system is that RFP-Cyto is likely sensitive to changes in NPC permeability^74–76^. Nup98 is an FG-Nup that regulates the NPC passive diffusion limit^77–79^. Surprisingly, depletion of NUP98 mRNA in our screen led to a decrease in the proportion of RFP-Cyto positive nuclei (Fig. 3A). Whether this result indicates that RFP-Cyto is insensitive to changes in NPC passive diffusion limits or whether this is due to the co-depletion of the core Nup Nup96, which is co-translated with Nup98, remains to be determined. These considerations indicate that live-cell imaging is still an important tool to validate increased nuclear rupture results.

A major gap in our understanding of nuclear membrane rupture is how the membrane itself responds to mechanical stress. Several lipid-based mechanisms have been identified that could relieve nuclear membrane tension and prevent nucleus rupture^18,19,80–86^, but whether these pathways are active during nuclear stress in mammalian cells is unknown. Our results support a role for membrane structural proteins in determining rupture frequency. Of the proteins driving GO:CC enrichment in nuclear/ER membrane categories, the majority regulate the physical structure of the membranes, including CTDNEP1, LNPK, Ndc1, and EVOLV1^67,87–95^.

CTDNEP1 regulates nuclear membrane amount and lipid composition through activation of the phosphatidic acid phosphatase, lipin1^67,80,89,90,92,96^, which has downstream effects on chromosome segregation, post-mitotic nuclear assembly, nuclear pore insertion, and protein stability^65,67,86,93,97^. CTDNEP1 may also regulate major signaling pathways, including myc, BMP and WNT, that can affect actin organization through lipin-independent mechanisms^98–104^. Our analysis of nuclear features after CTDNEP1 depletion demonstrates that the increase in membrane rupture is largely due to an unknown mechanism unlinked to nuclear lamina organization, actin bundle organization, or nuclear pore insertion defects. Defining the molecular mechanism of how CTDNEP1 regulates nuclear membrane stability, and its dependence on lipin1, could provide new information on how lipid composition contributes to nuclear membrane stress or how signaling pathways feedback to nuclear envelope structure.

In this study we developed several new tools that enable comprehensive identification of proteins and conditions that regulate nuclear membrane stability and their putative mechanism of action. This technology enabled us to identify 22 potential new rupture factors from a small-scale screen, and to demonstrate that one of these new factors, CTDNEP1, provides critical support for the nuclear membrane through a new mechanism potentially centered on lipid metabolism. This pipeline can be easily modified to identify specific rupture characteristics, including correlation with membrane blebs, or the integrity of other compartments, including micronuclei. Overall, the ability to rapidly quantify changes in nuclear rupture frequency using standard confocal microscopy will accelerate our understanding of the molecular mechanisms regulating nuclear envelope structure and dynamics and its consequences during development and disease.

## Methods

### Cell lines and transfection

U2OS (ATCC:HTB-96) and HeLa (ATCC:CCL-2) cells were grown in 1 × DMEM (GIBCO) plus 10% FBS (Sigma-Aldrich), 1% Penicillin–Streptomycin (GIBCO) at 10% CO_2_ and 5% CO_2_ respectively. U2OS shLMNB1 2 × RFP-NLS, U2OS 3 × GFP-NLS, and HeLa 3 × GFP-NLS cells were characterized previously^46^. siRNAs were transfected using siLentfect (Bio-Rad Laboratories) according to the manufacturer’s instructions and cells were analyzed at least 72 h post-transfection. RFP-Cyto was introduced by stable transduction into U2OS shLMNB1 GFP-NLS cells (RuptR) characterized previously^35^. U2OS cells were arrested in S phase by a single addition of 2 mM thymidine (Sigma-Aldrich), diluted fresh in PBS, 24 h before and during imaging. HeLa cells were arrested in S phase by a single addition of 2 mM hydroxyurea (EMD Millipore) diluted in water.

### Plasmids and siRNAs

1× and 2× RFP-luciferase constructs were made in pEGFP-C1 backbone by replacement of EGFP with TagRFP, then PCR and ligation of luciferase (1 × RFP-luciferase), then PCR and ligation of mCherry with C-terminal sequence N-terminal to TagRFP (2 × RFP-luciferase). pLVXE-Blast::mCherry-TagRFP-Luciferase (RFP-cyto) was constructed by PCR and ligation of mCherry-TagRFP-Luciferase from 2 × RFP-luciferase into a pLVXTight lentiviral vector (Clontech) previously modified to contain the EF1a promoter and blasticidin resistance.

Figure 1F,G siRNA validation: siRNAs against CTRL (non-targeting, cat#: D-001810-01-05), LMNA (5′-GGUGGUGACGAUCUGGGCUuu-3′), LMNB1 (5′-CGCGCUUGGUAGAGGUGGAUUuu-3′), and SUN1 (5′-ACCAGGUGCCUUCGAAAuu-3′) purchased from Horizon Discovery. Cells were left in 35 nM transfection medium for 48 h and assessed after 72 h.

siRNA screen: ON-TARGETplus SmartPool siRNAs were purchased in a 96-well plate from Horizon Discovery according to Table S9. U2OS cells were left in 35 nM transfection media 48 h and assessed after 72 h.

Single siRNA screen validation: ON-TARGETplus siRNAs were purchased from Horizon Discovery. siRNAs: CTRL (cat#: D-001810–01-05), LNPK (cat#: J-023148-09), RASA1 (cat#: J-005276-07), NRM (cat#: J-012779-22), ARID4A (cat#: J-003949-05), NDC1 (5′-CUGCACCACAGUAUUUAUAUU-3′), CTDNEP1 (cat#: J-017869–09), MYCBP2 (cat#: J-006951-05), BANF1 (5-AGUUUCUGGUGCUAAAGAAuu-3′), RB1 (5′- GAACAGGAGUGCACGGAUA-3′), STK11 (5′-UGAUGUGGUGCCGUACUU-3′). Individual siRNAs were transfected at 35 nM into U2OS shLMNB1 cells for 48 h and assessed after 72 h.

Figures 5, 6, S5: siRNAs were purchased from Horizon Discovery for CTDNEP1-9 (cat#: J-017869–09), and CTDNEP1-10 (cat#: J-017869–10). Control siRNAs are same as single siRNA screen validation. siRNAs were transfected at 50 nM in transfection medium and left on overnight (U2OS), or 5 h (HeLa).

### Fixed cell imaging

Samples fixed 45 m in 4% paraformaldehyde (Electron Microscopy Sciences) in PBS were stained 20 m with 1 µg/mL Hoechst (Life Technologies) and imaged as single confocal slices with a 40×/0.75 Plan Apo objective on a Leica DMi8 with a Yokogawa CSU spinning disc, Andor Borealis illumination, and an ASI automated stage with Piezo Z-axis. Images were captured with an Andor iXon Ultra 888 EMCCD camera using MetaMorph software (version 7.10.4; Molecular Devices) equipped with a plate acquisition journal. Acquisition parameters were set to capture 30 images per well. Images were corrected for background signal by mask image subtraction prior to intensity measurements and image displays exhibit rescaled intensity as noted in the CellProfiler 4.2.4 pipeline or modified for brightness/contrast in FIJI^99^. For fixed cell precision/recall analysis, cells were manually assessed for RFP-Cyto in the nucleus and results compared to the RFP + determinations made by the automated pipeline.

### Time-lapse imaging

Time-lapse images are single confocal slices captured with Leica DMi8 spinning disk microscope and 40×/0.75 Plan Apo objective, as described in Fixed Cell Imaging, at 3 m intervals over 18–24 h. Image sequences were adjusted for brightness and contrast using Fiji. For nuc:cyto mean intensity over time cell traces, nucleus and cytoplasm were manually segmented in Fiji for each time point. For live precision/recall analysis, true positives were manually determined by rapid loss of GFP-Nuc from the nucleus into the cytoplasm co-incident with rapid increase of RFP-Cyto in the nucleus.

### Immunofluorescence

Calreticulin: U2OS cells were grown on glass coverslips and fixed 10 m in methanol at − 20 °C and rehydrated 10 m in PBS at RT. Coverslips were blocked in 3% BSA in PBS plus 0.4% Triton X-100 for 30 m at RT and incubated in primary antibody rabbit anti–Calreticulin at 1:100 (Cell Signaling Technology) overnight at 4 °C. Goat anti rabbit Alexa Fluor 647 (Thermo Fisher Scientific) secondary antibody was diluted in blocking buffer 1:1000 and used for 30 m at RT. Coverslips were briefly incubated in 1 µg/mL DAPI (Roche) in PBS and mounted in Vectashield (Vector Laboratories). Actin: U2OS-shLMNB1-RFP-NLS cells were fixed 10 m in 4% PFA. Coverslips were blocked in 3% BSA in PBS plus 0.4% Triton X-100 for 30 min at RT and incubated in Alexa Fluor 488 Phalloidin (Cell Signaling Technology) at 1:1000 for 30 min. NUP133: U2OS GFP-NLS cells were fixed 5 m in 4% PFA. Coverslips were blocked in 3% BSA in PBS plus 0.4% Triton X-100 for 30 m at RT and incubated in primary antibody rabbit anti-Nup133 (Abcam) at 1:100 for 30 m. Goat anti rabbit Alexa Fluor 568 (Thermo Fisher Scientific) secondary antibody diluted in blocking buffer 1:2000 and used for 30 m at RT. Nuclear lamina: U2OS-shLMNB1-RFP-NLS cells were fixed 10 m in 4% PFA. Coverslips were blocked in 3% BSA in PBS plus 0.4% Triton X-100 for 30 min at RT and incubated in primary antibody mouse anti-laminA (Sigma Aldrich) at 1:500 overnight at 4 °C. Goat anti rabbit Alexa Fluor 488 (Thermo Fisher Scientific) secondary antibody diluted in blocking buffer 1:1000 and used for 30 m at RT.

Confocal z-stacks were acquired with a Leica TCS SP8 confocal microscope with a Leica HCX Plan Apo 63 ×/1.40 Oil CS2 objective with a z-step size of 0.15 µm. Post-acquisition, images were deconvolved using Lightning through the LAS × software. Images are single confocal sections. Images were adjusted for brightness and contrast using ImageJ.

### qRT-PCR

Cells were harvested from a confluent well in a 6-well plate with 0.25% Trypsin–EDTA, pelleted, and frozen at -80 °C. RNA was extracted using RNeasy Mini Plus kit (Qiagen) following manufacturer’s instruction. cDNA was synthesized using iScript RT Supermix (BioRad). A 5 ng/µL cDNA reaction mix was set up according to manufacturer’s instructions (SYBR green MM, Fisher Scientific). Primers were added at 150 nM except for STK11, which was added at 360 nM. Thermal cycling was performed at 50 °C for 2 min then 95 °C for 10 min then 40 cycles of 95 °C for 15 s, 60 °C for 60 s QuantStudio™ 5 Real-Time PCR System (ThermoFisher). Oligo sequences are listed in supplementary Table S23. Ct values for target genes were normalized internally to GAPDH and then to transcript levels in cells treated with non-targeting siRNA.

### Western blot

72 h after siRNA depletion and 24 h after S phase arrest, cells were lysed directly in 1 × LDS sample buffer (cat#: NP0007, Thermo Fisher) with β-mercaptoethanol. Proteins were separated in 4–20% precast polyacrylamide gels (cat#: 4561096, Bio-Rad) then transferred to nitrocellulose membrane. Prior to blocking and hybridization, membranes were cut so that longitudinal membrane sections were incubated with only one secondary antibody of each type (a maximum of two antibodies per section for Odyssey, a maximum of one antibody per section for HRP). Membranes were blocked in 5% milk:TBST then incubated 1 h RT with primary antibodies, washed, then incubated with secondary antibodies as noted below. Treated membranes were imaged on an Odyssey (LI-COR Biosciences), and bands were quantified by background subtracting the integrated density and normalizing to tubulin bands using Fiji/ImageJ. Primary antibodies: LMNA (1:1000, cat#: L1293, Sigma Aldrich), LMNB1 (1:1000, cat#: sc-365214, Santa Cruz), SUN1 (1:1000, cat#: NBP1-87396, Novus Biologicals), tubulin (1:1000, cat#: 3873S, Cell Signaling Technology) Secondary antibodies: 680- or 790-conjugated Alexa Fluor (1:10,000, cat#: A10043 and A11371, Thermo Fisher). For the BAF immunoblot: blocked membranes were incubated for 24 h in anti BAF (1:250, cat# sc-166324, Santa Cruz), washed, and incubated for 45 min at room temp. in goat anti-mouse HRP (1:5000, cat# G21040, Life Technologies). Membranes were washed for 1 h at 4 °C, incubated in SuperSignal West Femto Chemiluminescent Substrate (Fisher Scientific), and imaged, along with tubulin control, on a ChemiDoc Imaging System (Bio-Rad).

### NUP133 localization analysis

Cells were imaged with a 63×/1.40 objective with 0.2 µm z step and processed by Lightning deconvolution (Leica). In Imaris, surfaces were created based on DAPI signal. All cutoff or missegmented nuclei were manually removed from analysis. Spots were created on NUP133 channel with estimated diameter of 0.2 µm xy and 0.44 um, z, and filtered based on quality and median intensity to minimize noise on a top section of the dimmest nucleus in each image.

### Lamina gap analysis

Cells were imaged with a 63×/1.40 objective with 0.2 µm z-step and processed with lightning deconvolution (Leica). An app was created in MATLAB (R2022b) for the quantification and characterization of lamina gaps. The algorithm that we developed to identify gaps in the 3D nuclear envelope relies on the comparison between the regular, cartesian distance transform map, and the geodesic distance transform map. The rationale is that the geodesic distance between a given pixel and a reference point is the same as the regular, cartesian distance only if the path is not constrained (i.e., a gap in the lamina). Finding equality between the two distance maps will thus reveal the gap areas, akin to rays of light coming out of a punctured sphere containing an illumination source. These rays of light are then projected onto the contour of the nucleus (defined by the perimeter of the DAPI signal) to extract the precise size and location of the gaps.

Briefly, Lamin and DAPI signals were segmented in 3D using standard procedures. After a slight dilation of the nucleus (DAPI) object, the perimeter pixels of the resulting object were defined. Erosion of the DAPI object was used as a reference/ seed for distance transform computations, and the complement of the binarized lamin signal was used to constrain the geodesic distance computation. The intersect between the dilated DAPI perimeter and the distance equality map defines the lamina gaps. Mis-segmented nuclei were removed from analysis based on nuclear volume and negative curvature of gaps. Gaps over 5 μm^3^ were manually verified and resulted from merging multiple gaps nearby. These gaps were removed from analysis.

To quantify the local nuclear curvature associated with lamin gaps, we created a bounding volume (alphashape) that envelops the binarized DAPI signal with a cloud density of 10 points per µm^2^. Position, surface normals and curvature values were then extracted for each point using a 3-by-3 kernel. This analysis was implemented by the 'findPointNormals()' function^100^. Curvature values covering a given gap were then averaged.

### Statistics and quantification

Quantification of fixed image data generated by CellProfiler 4.2.4: Images corrected for background signal were analyzed for mean intensity of RFP and GFP in the nucleus and cytoplasm using mask objects generated by rescaling intensity, down-sampling, median filtering, and adaptive Otsu thresholding of Hoechst (nucleus) and RFP (cytoplasm). Morphological measurements of the nucleus used for data filtering and comparisons were generated from the Hoechst mask. Rupture frequency for fixed images was determined either by data output from CellProfiler, in which rupture was determined if the nuc:cyto ratio of RFP-Cyto exceeded one standard deviation above the median for the experimental control (RFP +), cyto:nuc ratio of GFP-Nuc exceeded one standard deviation above the median for the experimental control (GFP +), or manually (as noted for validation) based on visual identification of nuclear RFP signal. Intensity outputs from CellProfiler were measured from eroded masks to reduce error from over-segmentation.

Quantification of rupture frequency and duration in time-lapse image analysis was done manually in ImageJ by visually identifying a rapid loss of compartmentalization and subsequent restoration of nuclear localization signal, as previously implemented^46^.

Hypergeometric enrichment analysis was completed in R 4.2.1 using the mysigdbr and clusterProfiler packages. For enrichment of statistically significant rupture frequency hits within the context of the screen, gene sets from the Broad Institute C5:GO database were first joined to the complete gene list for the siRNA screen, ensuring hit enrichment calculations were performed relative to the landscape of the screen. Enrichment scores were determined by dividing the gene ratio by the background ratio, and p-values determined by Fishers exact test as generated by the enricher function (enricher (gene = hits, TERM2GENE = screen)).

All statistical tests were conducted using R (version 4.2.1). Each dataset was completed with a minimum of N = 3 biological replicates. Pair-wise comparisons on categorical data were analyzed using Barnard’s exact test (“Barnard” package, R). Bonferroni correction for multiple testing applied as indicated for statistical analyses with greater than five comparisons. Pairwise comparisons on continuous data used Wilcoxon rank sum test (base R) or Kolmogorov–Smirnov test (base R) and comparisons between more than 2 groups were evaluated with Kruskal–Wallis family test followed by Dunn's Multiple Comparison correction. Tables for statistical analyses are included as tabs in the Supplementary Tables spreadsheet.

### Supplementary Information

Supplementary Figure 1.

Supplementary Figure 2.

Supplementary Figure 3.

Supplementary Figure 4.

Supplementary Figure 5.

Supplementary Figure 6.

Supplementary Tables.

Supplementary Video 1.

Supplementary Video 2.

Supplementary Video 3.

Supplementary Video 4.

Supplementary Video 5.

Supplementary Video 6.

Supplementary Legends.

Supplementary Legends.

## Data Availability

The image datasets analyzed in the current study are available from the corresponding author on reasonable request. All software and analysis R code is freely available on our github page: https://github.com/hatch-lab.

## Abbreviations

53BP1: P53 binding protein 1
BANF1: Barrier to autointegration factor
cGAS: Cyclic GMP-AMP synthase
CTDNEP1: CTD nuclear envelope phosphatase 1
GO: Gene ontology
HSP90: Heat shock protein 90
NES: Nuclear export signal
NLS: Nuclear localization signal
PML bodies: Promyelocytic leukemia bodies
