# Supplementary material for: A high-content screen reveals new regulators of nuclear membrane stability
Source: Sci Rep. 2024 Mar 12;14:6013. doi: 10.1038/s41598-024-56613-1 (PMC10933478; doi:10.1038/s41598-024-56613-1)
Supplement: Supplementary file 1 — Supplementary Figure 1. [file 41598_2024_56613_MOESM1_ESM.pdf]

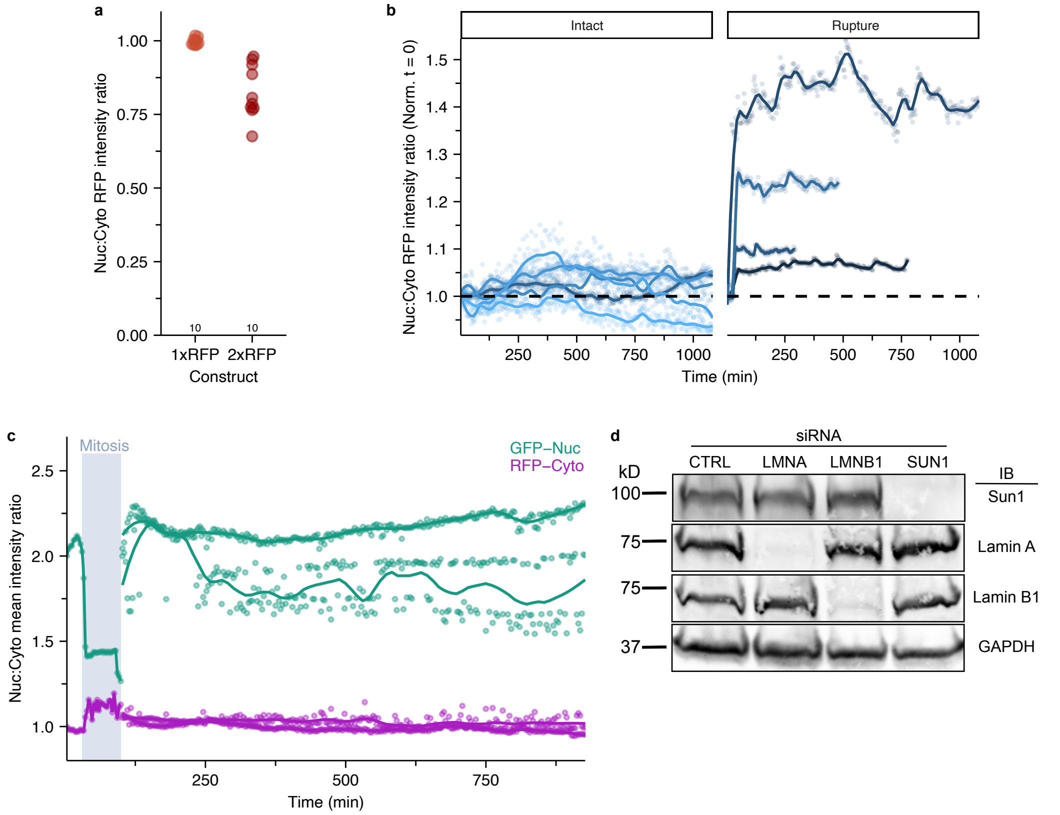

Figure S1. a. Comparison of Nuc:Cyto RFP mean intensity ratio for 1x vs 2xRFP RFP-Cyto constructs after transfection in U2OS shLMNB1 GFP-Nuc cells. 1xRFP shows similar intensities in nucleus and cytoplasm and 2xRFP shows reduced nucleus intensity. N =1. **b.** Traces of 4 cells undergoing at least one nucleus rupture (Rupture) and 5 cells with no nucleus ruptures (Intact), determined by GFP-Nuc mislocalization, show sustained increase in nuclear RFP signal only after nucleus rupture. N = 2. **c.** Representative trace of Nuc:Cyto mean intensity for RFP-Cyto and GFP-Nuc prior to, during, and after mitosis (high GFP-Nuc in cytoplasm) during live-cell imaging. RFP-Cyto is rapidly excluded from the nucleus after mitosis, defined as the onset of GFP-Nuc accumulation in nucleus. N = 1. **d.** Western blot indicating efficacy of positive and negative control single siRNA knockdowns. Cells = U2OS RuptR unless indicated. n values indicated on graphs.
