# Supplementary material for: A high-content screen reveals new regulators of nuclear membrane stability
Source: Sci Rep. 2024 Mar 12;14:6013. doi: 10.1038/s41598-024-56613-1 (PMC10933478; doi:10.1038/s41598-024-56613-1)
Supplement: Supplementary file 2 — Supplementary Figure 2. [file 41598_2024_56613_MOESM2_ESM.pdf]

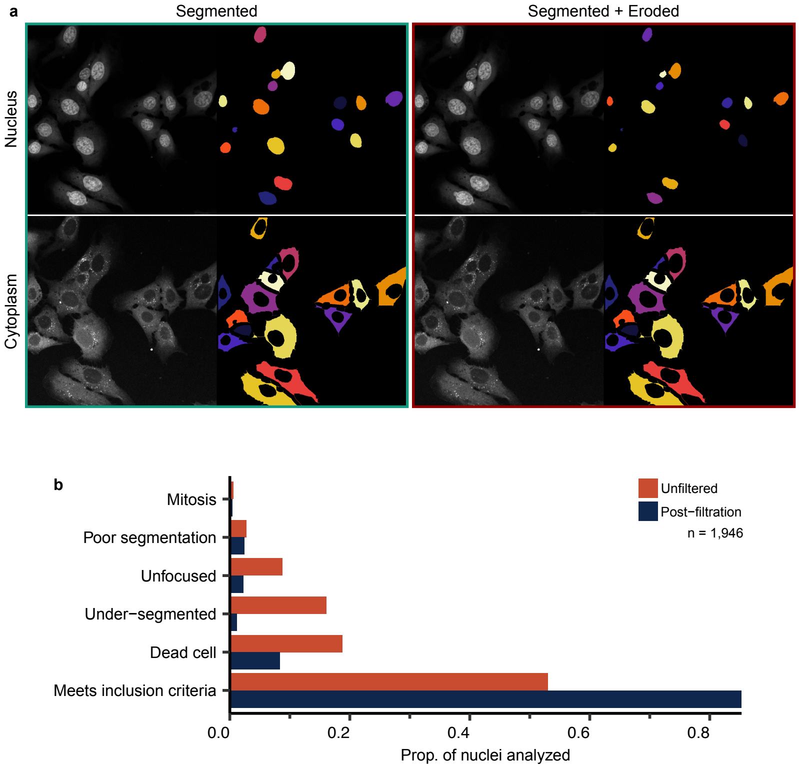

Figure S2. a. Representative images of the initial nucleus and cytoplasm segmentation, used for morphology analyses (left), and the eroded objects, used for mean fluorescence intensity measurements (right). **b.** Proportion of nuclei manually annotated as properly segmented and analyzable before and after applying morphology and intensity filters during the analysis pipeline. Post-filtration, all classes of contaminating cell types are reduced and the proportion of cells that meet all the inclusion criteria for analysis are increased. Images likely enriched in missegmentation classes were selected from multiple screen wells from a single replicate.
