# Supplementary material for: A high-content screen reveals new regulators of nuclear membrane stability
Source: Sci Rep. 2024 Mar 12;14:6013. doi: 10.1038/s41598-024-56613-1 (PMC10933478; doi:10.1038/s41598-024-56613-1)
Supplement: Supplementary file 3 — Supplementary Figure 3. [file 41598_2024_56613_MOESM3_ESM.pdf]

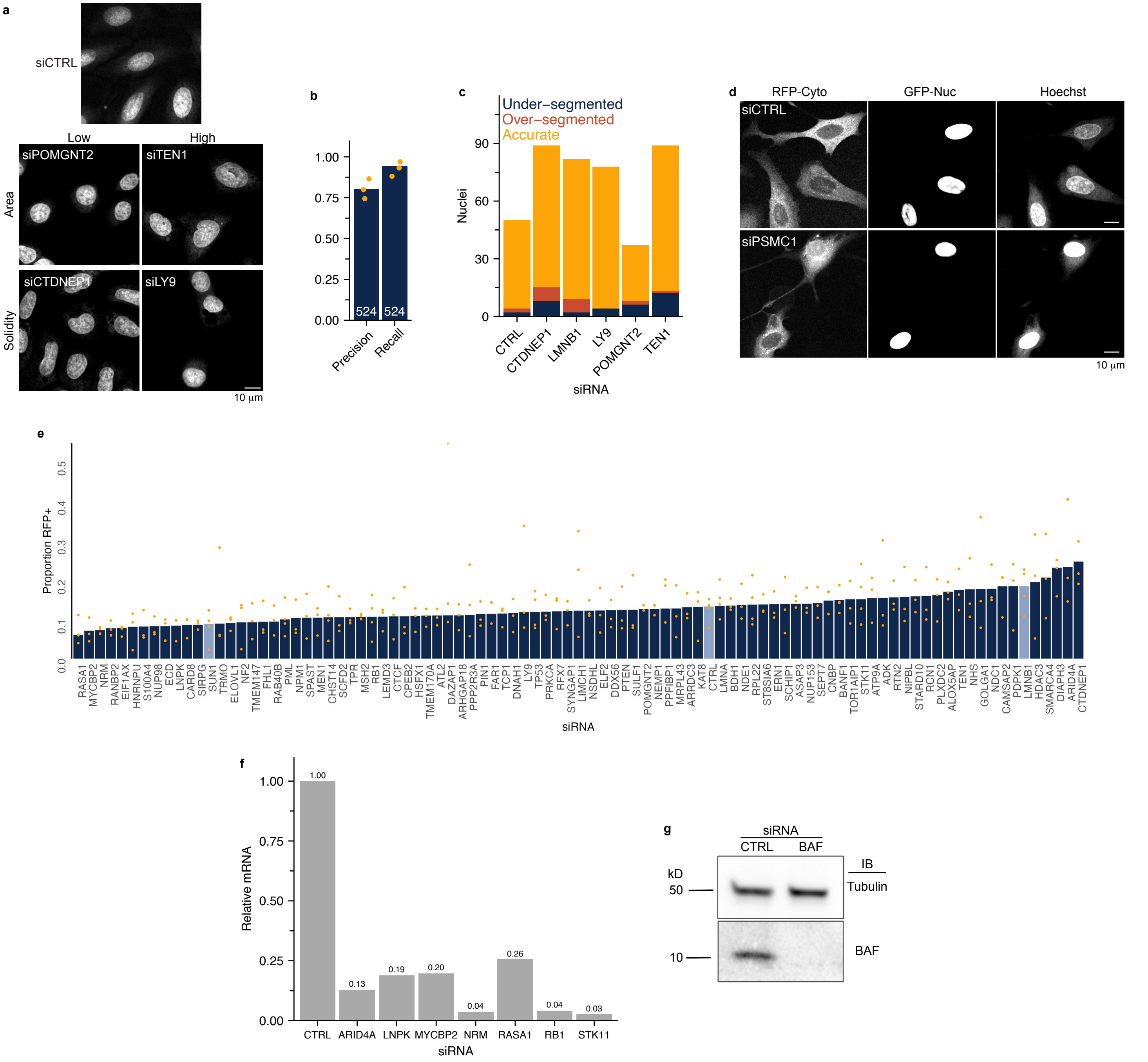

Figure S3. a. Representative images (Hoechst) of morphology screen hits. **b.** Manual precision/recall analysis of RFP-Cyto positive nuclei calls on images from control siRNA wells and morphology screen hits. N = 3. **c.** Breakdown of segmentation fidelity for images analyzed in (b). **d.** Representative images of control and siPSMC1 wells showing evidence of cell death from PSMC1 depletion, including cell rounding and increased Hoechst intensity. **e.** Full screen results showing fold change Nuc:Cyto RFP-Cyto ratio values over filter threshold (RFP+). N = 4. **f.** qRT-PCR analysis of mRNA depletion by single siRNA transfection for targets assessed in Fig. 3B and D. N = 1. Calculated values shown on graph. **g.** Western blot analysis of protein depletion by single BAF siRNA transfection. Cells: U2OS RuptR.
