# Supplementary material for: A high-content screen reveals new regulators of nuclear membrane stability
Source: Sci Rep. 2024 Mar 12;14:6013. doi: 10.1038/s41598-024-56613-1 (PMC10933478; doi:10.1038/s41598-024-56613-1)
Supplement: Supplementary file 4 — Supplementary Figure 4. [file 41598_2024_56613_MOESM4_ESM.pdf]

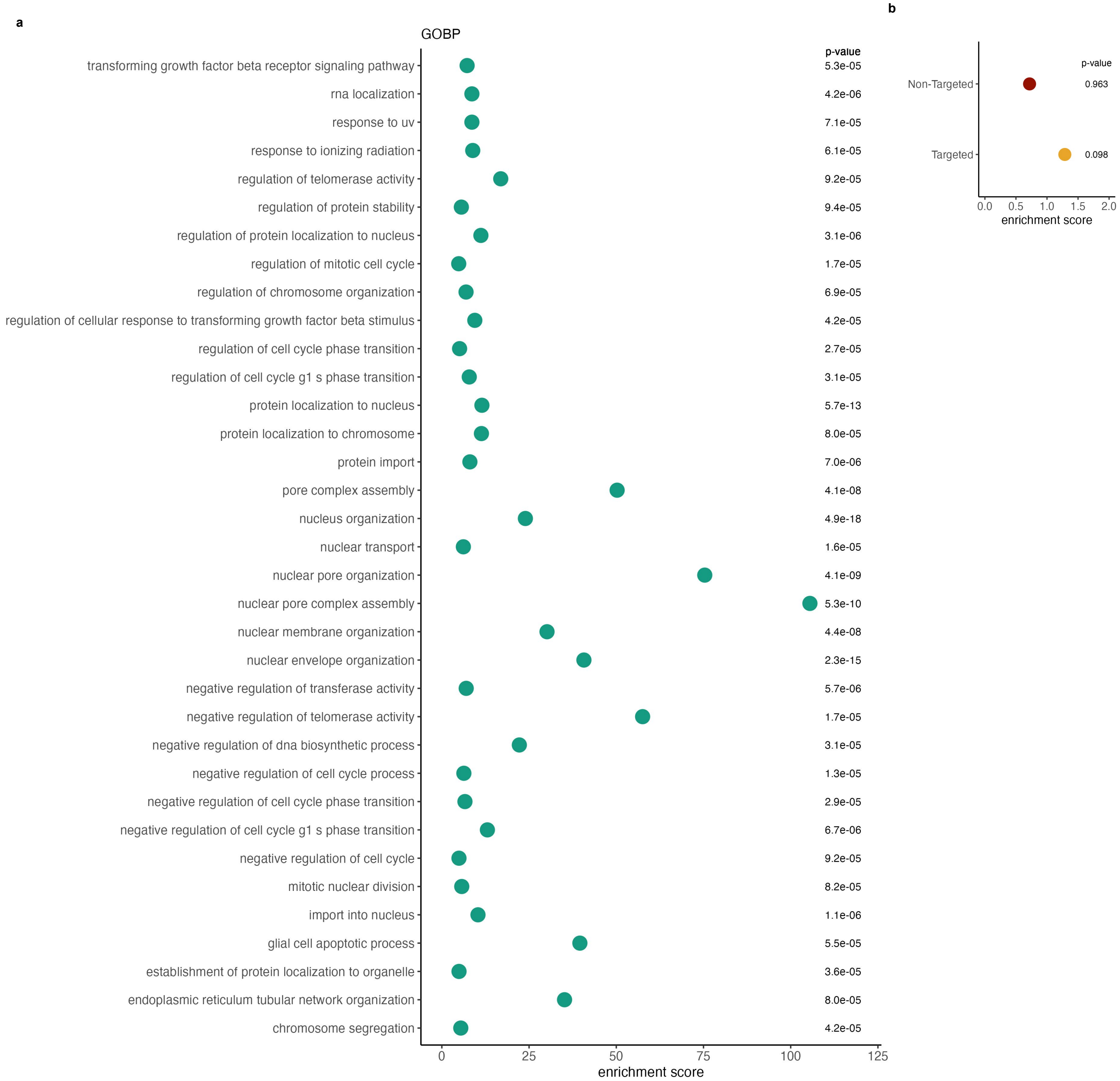

Figure S4. a. Enrichment of all siRNAs included in the screen relative to the GOBP gene set, p-value indicated on the right. **b.** Hypergeometric enrichment finds a bias towards, but not significant enrichment of siRNAs specifically selected for the screen in the screen hits. Stats: Table S12-13.
