# Supplementary material for: A high-content screen reveals new regulators of nuclear membrane stability
Source: Sci Rep. 2024 Mar 12;14:6013. doi: 10.1038/s41598-024-56613-1 (PMC10933478; doi:10.1038/s41598-024-56613-1)
Supplement: Supplementary file 5 — Supplementary Figure 5. [file 41598_2024_56613_MOESM5_ESM.pdf]

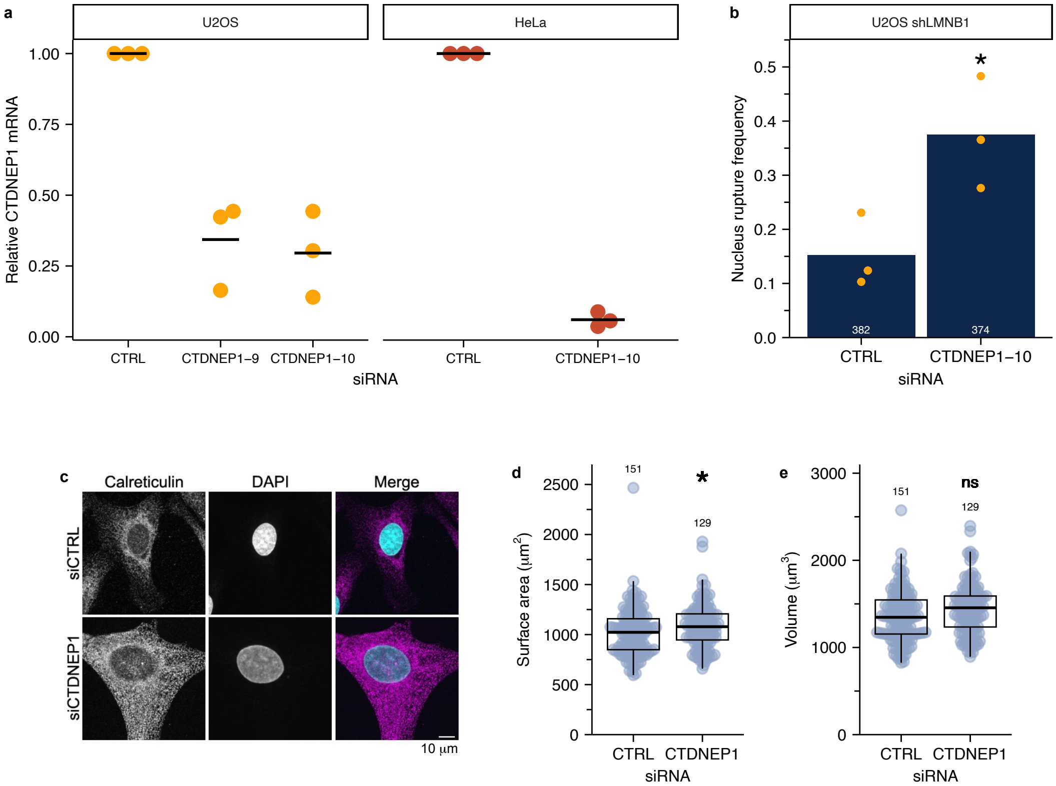

Figure S5. a. qRT-PCR results for CTDNEP1 siRNAs (9 and 10) in U2OS and HeLa cells expressing GFP-Nuc. Line = mean. **b.** Rupture frequency increases following siRNA depletion of CTDNEP1 using a second single siRNA (CTDNEP1-10) analyzed by live-cell imaging. * = $p < 0.05$, Barnards test. **c.** Representative images showing increased ER levels (calreticulin) in CTDNEP1 depleted cells. **d-e.** Distribution of nucleus surface area (d) and volume (e) in siRNA treated cells. * = $p < 0.05$, ns = $p > 0.05$, K-S test. Cells: U2OS shLMNB1 2x RFP-NLS unless otherwise noted. Stats: Table S17, S19. N = 3 for all quantifications.
