# Supplementary material for: A high-content screen reveals new regulators of nuclear membrane stability
Source: Sci Rep. 2024 Mar 12;14:6013. doi: 10.1038/s41598-024-56613-1 (PMC10933478; doi:10.1038/s41598-024-56613-1)
Supplement: Supplementary file 6 — Supplementary Figure 6. [file 41598_2024_56613_MOESM6_ESM.pdf]

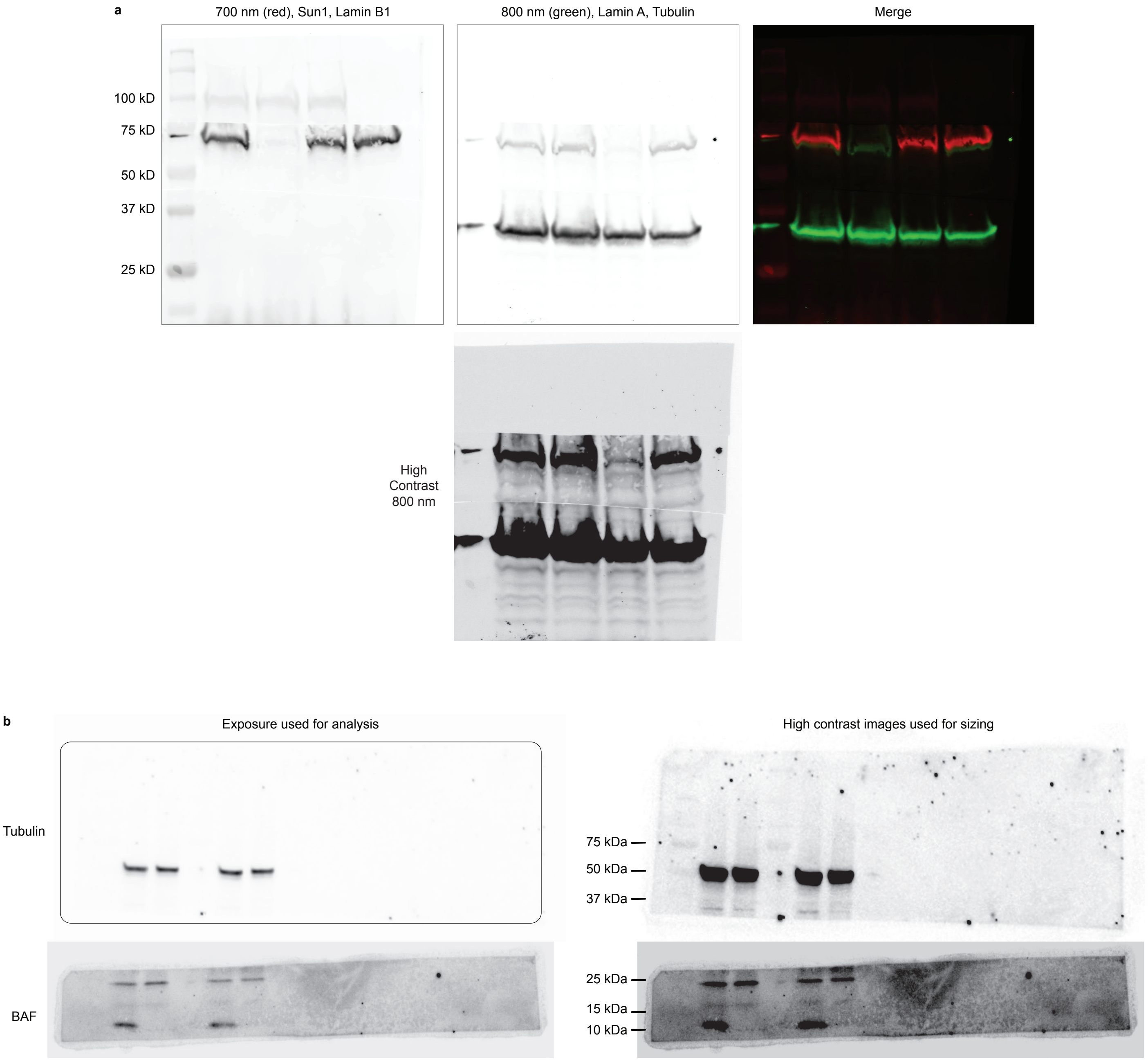

Figure S6. Original Western blots. a. Full immunoblot imaged on multichannel fluorescent imager, single exposure, related to figure S1d. Membrane cut longitudinally prior to hybridiziation and blot segmented laterally to limit hybridization to used lanes. Individual channels (inverted) and merge are shown. Blot was aligned with imager edges on two sides, leading to loss of blot edge imaging. **b.** Full immunoblot imaged on chemiluminescence imager, related to figure S3g. Membrane cut prior to hybridization. BAF and tubulin membranes imaged independently with independently adjusted exposures. Approximate area of tubulin blot demarcated by a line on analysis image. Two technical replicates present on blot, only the first one shown in figure S3g.
