# Supplementary material for: A high-content screen reveals new regulators of nuclear membrane stability
Source: Sci Rep. 2024 Mar 12;14:6013. doi: 10.1038/s41598-024-56613-1 (PMC10933478; doi:10.1038/s41598-024-56613-1)
Supplement: Supplementary file 14 — Supplementary Legends. [file 41598_2024_56613_MOESM14_ESM.docx]

**Supplementary Movies**

**Movie S1.** Shown are a rupturing (right) and non-rupturing (left) U2OS RuptR cell. Channel order = RFP-Cyto, GFP-Nuc, merge. Scale bar = 10 um. Time stamp = hh:mm. Movie speed = 7 fps.

**Movie S2.** Shown is a mitotic U2OS RuptR cell, channel order RFP-Cyto, GFP-Nuc, merge. Scale bar = 10 um, time stamp = hh:mm. Movie speed = 7 fps.

**Movie S3.** False negative U2OS RuptR cell, with rupture occurring at 11 s. Although there is a slight increase in nucleus RFP, the mean intensity did not meet the threshold for this single frame rupture. Channel order = RFP-Cyto, GFP-Nuc, merge. Scale bar = 10 um. Time stamp = hh:mm. Movie speed = 1 fps.

**Movie S4.** False Positive U2OS RuptR cell. Channel order = RFP-Cyto, GFP-Nuc, merge. Scale bar = 10 um. Time stamp = hh:mm. Movie speed = 7 fps.

**Movie S5.** U2OS RuptR cell, with single frame small rupture occurring at 11 s. Channel order = RFP-Cyto, GFP-Nuc, merge. Scale bar = 10 um. Time stamp = hh:mm. Movie speed = 1 fps.

**Movie S6.** Shown are nuclear ruptures in U2OS shRNA-LMNB1 2xRFP-NLS cells transfected with siCTRL (left) and siCTDNEP1 (right) siRNAs. Channel = RFP-NLS. Scale bar = 10 um, time stamp = hh:mm, Movie speed = 7 fps.
