# Supplementary material for: A high-content screen reveals new regulators of nuclear membrane stability
Source: Sci Rep. 2024 Mar 12;14:6013. doi: 10.1038/s41598-024-56613-1 (PMC10933478; doi:10.1038/s41598-024-56613-1)
Supplement: Supplementary file 15 — Supplementary Legends. [file 41598_2024_56613_MOESM15_ESM.docx]

**Supplementary Tables**

Table S1. Statistics associated with Fig. 1G, live vs fixed cell analysis of RuptR

Table S2. Statistics associated with Fig. 2D, known siRNA pipeline validation

Table S3. Statistics associated with Fig. 3A, siRNA screen RFP-Cyto intensity analysis

Table S4. Statistics associated with Fig. 3B, RFP-Cyto hits validation

Table S5. Statistics associated with Fig. 3C, siRNA screen GFP-Nuc intensity analysis

Table S6. Statistics associated with Fig. 3D, GFP-Nuc hits validation

Table S7. Statistics associated with Fig. 3E, siRNA screen nucleus area analysis

Table S8. Statistics associated with Fig. 3F, siRNA screen nucleus solidity analysis

Table S9. Table of siRNAs included in screen

Table S10. Statistics associated with Fig. 4A, hypergeometric enrichment GOBP

Table S11. Statistics associated with Fig. 4B, hypergeometric enrichment GOCC

Table S12. Statistics associated with Fig. S4A, hypergeometric enrichment screen bias

Table S13. Statistics associated with Fig. S4B, hypergeometric enrichment targeted vs random

Table S14. Statistics associated with Fig. 5A, CTDNEP1 rupture frequency analysis

Table S15. Statistics associated with Fig. 5B, CTDNEP1 rupture duration analysis

Table S16. Statistics associated with Fig. 5C, CTDNEP1 micronucleus rupture analysis

Table S17. Statistics associated with Fig. 5E, S5D, S5E, CTDNEP1 nucleus morphology analysis

Table S18. Statistics associated with Fig. 5H, CTDNEP1 Nup133 analysis

Table S19. Statistics associated with Fig. S5B, CTDNEP1-10 rupture frequency analysis

Table S20. Statistics associated with Fig. 6C, CTDNEP1 nuclear lamina gap proportions

Table S21. Statistics associated with Fig. 6D, 6E, CTDNEP1 nuclear lamina gap volume and number

Table S22. Statistics associated with Fig. 6F, CTDNEP1 nuclear curvature at lamina gap

Table S23. List of qRT-PCR primers used in study.
